# Signal pathways involved in contrast-induced acute kidney injury

**DOI:** 10.3389/fphys.2024.1490725

**Published:** 2024-11-25

**Authors:** Ke Deng, Mingxin Pei, Beibei Li, Nanqi Yang, Zijian Wang, Xinchi Wan, Zhiying Zhong, Zhiyi Yang, Yanling Chen

**Affiliations:** Department of Pathophysiology, Zhuhai Campus of Zunyi Medical University, Zhuhai, Guangdong, China

**Keywords:** contrast-induced acute kidney injury, signal pathways, oxidative stress, inflammation, apoptosis, ferroptosis

## Abstract

Contrast-induced acute kidney injury (CI-AKI) has emerged as a global public health concern, ranking as the third most prevalent cause of hospital-acquired acute kidney injury, which is related to adverse outcomes. However, its precise pathogenesis remains elusive. Consequently, researchers are dedicated to uncovering CI-AKI’s pathophysiology and signaling pathways, including inflammation, oxidative stress, apoptosis, and ferroptosis, to improve prevention and treatment. This review thoroughly analyzes the signaling pathways and their interactions associated with CI-AKI, assesses the impact of various research models on pathway analysis, and explores more precise targeted treatment and prevention approaches. Aims to furnish a robust theoretical foundation for the molecular mechanisms underpinning clinical treatments.

## 1 Introduction

In recent years, rapid advancements in radiology and interventional cardiology have increasingly affected the disease diagnosis and treatment. Consequently, the widespread utilization of contrast medium (CM) has become commonplace (Huang et al., 2022). CI-AKI is defined as an increase in serum creatinine ≥25% or ≥0.5 mg/dL (44 μmol/L) above baseline within 48–72 h after intravenous administration of CM, with exclusion of other causes of renal impairment (Liu et al., 2019). Notably, CI-AKI significantly impedes patient recovery, prolongs hospitalization, escalates hospital-related costs, and increases the risk of chronic kidney disease (Oh et al., 2019). According to statistics, CI-AKI is the third most common form of hospital-acquired acute kidney injury, following renal insufficiency due to poor perfusion and nephrotoxic drugs. Some studies have indicated that 11%–40% of patients who receive CM injections will develop CI-AKI (Li and Wang, 2024). The incidence of CI-AKI varies with the patient’s condition; the incidence rate in patients with normal renal function is 5%, whereas it increases to 20% in high-risk patients, such as the elderly, those with renal insufficiency, diabetes mellitus, cardiac insufficiency, and those exposed to nephrotoxic drugs, among other factors (Iranirad et al., 2024). In more serious cases, such as in patients with combined renal insufficiency and diabetes, the incidence of CI-AKI may be as high as 38%. These data indicate that CI-AKI is a noteworthy issue, especially among high-risk patient groups. To reduce the incidence of CI-AKI, it is essential to conduct a thorough risk assessment of patients and to implement appropriate preventive measures before administering CM.

**FIGURE 1:**
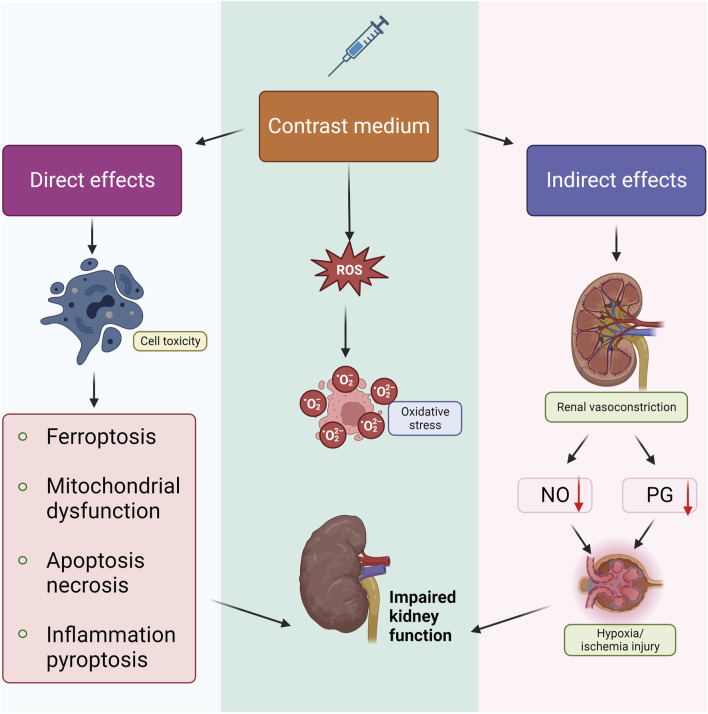
Pathophysiological mechanism of CI-AKI. There are three main aspects of the mechanism of CI-AKI genesis that are currently generally recognized. Firstly, CM can cause apoptosis, necrosis, pyroptosis, inflammatory response, and ferroptosis through direct cytotoxicity; in addition, CM promotes the generation and release of ROS and thus leads to oxidative stress; and CM can cause renal vasoconstriction and inhibit the production of NO, PG, and other vasodilating active ingredients, which leads to ischemia and hypoxia injury, and ultimately leads to renal impairment in combination with the other two mechanisms.

The pathogenesis of CI-AKI is intricate, involving multiple pathophysiologic processes. Research indicates that renal medullary ischemia and hypoxia, direct tubular cytotoxicity of CM, and mitochondrial dysfunction all contribute to its development. Additionally, the overproduction of reactive oxygen species (ROS) plays a role. The mechanism of CI-AKI involves three major aspects: direct effects, indirect effects, and ROS production (Kusirisin et al., 2020) (Figure 1). Direct effects include CM-induced damage to renal cells, including renal tubular epithelial and endothelial cells, leading to mitochondrial dysfunction, apoptosis or necrosis, and inflammation (Mehran et al., 2019). Indirect effects involve alterations in renal hemodynamics, causing intrarenal vasoconstriction and medullary ischemia and hypoxia. This is coupled with a reduction in the synthesis of vasodilatory factors such as nitric oxide (NO) and prostaglandins (PG), which exacerbates medullary ischemia and hypoxia (Li and Pan, 2022). CM induces overproduction of ROS and diminishes antioxidant enzyme activity, leading to heightened oxidative stress and impaired renal function. Despite extensive research efforts, the complete elucidation of CI-AKI pathology has remained elusive. Numerous adjunctive drug therapies have been explored for CI-AKI prevention, yet few have effectively halted its progression (Luo et al., 2022). A comprehensive understanding of the molecular mechanisms behind CI-AKI is essential to mitigate the adverse outcomes of high-risk CI-AKI patients. Therefore, a thorough exploration of the signaling pathways involved in CI-AKI is imperative. This review deeply explores the impact of different models on CI-AKI pathway research, as well as the main signaling pathways identified in previous studies and further clarifies their roles in the onset and progression of CI-AKI and their potential compensatory mechanisms. Its findings have important clinical significance for the development of effective prevention and treatment strategies.

## 2 Models

### 2.1 Cell models

*In vitro* models serve as the cornerstone of pathway research, allowing researchers to precisely control experimental conditions and biochemical stimuli while eliminating environmental interference. They also permit the precise manipulation of molecules and signaling pathways, investigating their functions and mechanisms, thus providing scientific support for understanding diseases and developing new drugs.

In CI-AKI cell model research, monitoring the changes in pathway indicators after cell exposure to CM is one of the fundamental methods for exploring signaling pathways. These changes include, but are not limited to, adjustments in protein and gene expression levels (Guo et al., 2021). Additionally, by combining the use of pathway inhibitors and agonists, the function of specific pathways in CI-AKI can be revealed. Lei R’s research has shown that CM treatment can trigger the production and damage of mitochondrial ROS, and the application of autophagy activators can effectively protect HK-2 cells from contrast agent-induced apoptosis (Lei R. et al., 2018). Conversely, autophagy inhibitors exacerbate cell damage, indicating that the mitochondrial autophagy pathway plays a crucial protective role in CI-AKI. At the same time, the use of small interfering RNA (siRNA) transfection technology to construct an *in vitro* model has also provided a powerful scientific tool for signaling pathway research. In the study by Li Y’s team, HK-2 cells transfected with NOD-like receptor family pyrin domain-containing 3 (NLRP3) and Cysteine-aspartate protease 1 (Caspase-1) specific siRNA showed that CM affects the pyroptosis process of renal tubular epithelial cells through the ROS/NLRP3/Caspase-1/Gasdermin D signaling pathway, and inhibiting these pathways can effectively alleviate CM-induced cell damage (Li et al., 2022).

However, *in vitro* models typically only include specific cell types, which lack the complexity of the *in vivo* environment, such as the interactions between multiple cell types. This may lead to simplification or deviation of the pathway function.

### 2.2 Animal models

*In vivo* models are vital for pathway research, accurately mimicking physiological and pathological states with a complete signaling system, crucial for complex pathways involving multiple cell types. They also serve as a platform to study drug absorption, distribution, and metabolism, essential for understanding drug effects on specific pathways.

A common approach to studying signaling pathways in CI-AKI is to use animal models, monitoring pathway indicators. The model construction typically involves injecting indomethacin and Nω-nitro-L-arginine methyl ester (L-NAME) followed by a contrast agent to induce kidney damage (Deng K. et al., 2023; Xiang et al., 2024). This method is favored for its ability to enhance the nephrotoxic effects of contrast agents by disrupting renal blood flow. However, it may complicate mechanism interpretation due to the drugs’ effects on PG, NO, and related pathways. Another method involves dehydrating rats before CM exposure or using partial nephrectomy or diabetic models to simulate pre-existing renal conditions (Huang et al., 2020; Wang et al., 2024a). Researchers choose the most appropriate model for their specific research needs. Utilizing existing CI-AKI animal models in combination with gene knockout or gene knockdown techniques allows for more accurate study of the mechanisms of signaling pathways. Through gene knockout, researchers can directly observe the impact of gene function loss on CI-AKI and infer the role of the gene in specific pathways. For instance, Zhang Z and colleagues have revealed the key role of *Caspase-11* in CI-AKI (Zhang Z. et al., 2018). Gene knockdown technology can reduce gene expression to observe the impact of partial function loss on pathways. Compared to gene knockout, gene knockdown is typically reversible, which allows researchers to explore the effects of gene expression changes on pathways at different time points and study how changes in gene expression levels affect other genes or proteins, thereby uncovering complex regulatory networks. Studies that reduce the expression of the *Nrf2* gene have confirmed its key protective role in CI-AKI (Ran et al., 2022). Through gene knockout or knockdown techniques, we can gain a deeper understanding of the roles of these key genes in pathways, which not only helps to reveal the pathological mechanisms of CI-AKI but also provides the possibility of discovering new drug targets, thereby offering more effective strategies for the prevention and treatment of CI-AKI.

*In vitro* and *in vivo* models are complementary in research, with the former suited for initial screening and mechanism studies, and the latter for validation and whole-organism effect assessment. Transitioning from *in vitro* to *in vivo* models facilitates the application of research findings in clinical settings. Model choice is determined by research objectives and challenges.

## 3 Inflammatory response pathways

The inflammatory response is vital for tissue homeostasis and protection against infection and injury, but its dysregulation can cause chronic infections, sepsis, and organ failure (Liu Y. et al., 2023). In CI-AKI, inflammation is a complex pathogenic factor, with high inflammatory factor levels indicating risk (Alshogran et al., 2022). CM induces oxidative stress, generating ROS that drive lipid peroxidation, inflammation, vascular injury, and hypoxia, creating a feedback loop that fosters CI-AKI development.

### 3.1 NLRP3 pathway

Research has demonstrated the pivotal role of NLRP3 as a crucial inflammatory molecule in the body’s immune response and the development of diseases. It functions prominently as a component of innate immunity (Zhang et al., 2023). The NLRP3 inflammasome comprises: NLRP3, an apoptosis-associated speck-like protein containing a CARD (ASC), and the effector protein Caspase-1. The intricate interactions among these proteins tightly regulate inflammasome function, ensuring its immunological activity only at the appropriate time (Biasizzo and Kopitar-Jerala, 2020). Pyroptosis, a form of programmed lytic cell death triggered by inflammatory caspases, is activated when the stimulus signal binds to NLRP3, promoting the formation of inflammatory vesicles and activating Caspase-1. Activated Caspase-1 induces the secretion of Interleukin (IL)-1β and IL-18 by cleaving pro-IL-1β and pro-IL-18, thereby mediating the inflammatory response and ultimately leading to cellular pyroptosis (Zeng et al., 2022; Lei Q. et al., 2018). Studies have indicated that systemic exposure to CM induces pyroptosis in renal tubular epithelial cells (Chen F. et al., 2020; Li et al., 2022). The NLRP3 inflammasome proves indispensable for the development of CI-AKI, with its pathway activated during acute tubular injury, actively participating in local inflammation during CI-AKI (Lau et al., 2018). In a mouse model of CI-AKI, the NLRP3 inflammasome was observed to be activated in renal tissues, and the knockdown of *nlrp3* mitigated CM-induced renal injury and apoptosis in mice (Shen et al., 2016).

Toll-like receptor 4 (TLR4) emerges as a key player in inflammatory signaling, contributing to the development of renal interstitial inflammation. CI-AKI studies reveal that CM-induced activation of TLR4 triggers the immune and inflammatory response by regulating downstream signaling factors, namely NLRP3 and Nuclear factor kappa-light-chain-enhancer of activated B cells (NF-κB), through the Myeloid differentiation primary response 88(MyD88)-dependent pathway. This cascade, in turn, releases a plethora of inflammatory cytokines and effector cell molecules, ultimately resulting in renal inflammation (Yue et al., 2017; Tan et al., 2017). Further investigations suggest that statins may inhibit CM-induced inflammation and oxidative stress while safeguarding renal function through targeted inhibition of TLR4 expression and specific regulation of the TLR4/MyD88 signaling pathway (Yue et al., 2017). Moreover, in a prior study, salvianolic acid B was found to suppress inflammation and attenuate CI-AKI by inhibiting the TLR4/NF-κB/NLRP3 signaling pathway (Pei et al., 2022). These collective findings underscore the critical role of NLRP3 inflammatory vesicles and their associated pathways in the inflammatory response to CI-AKI.

### 3.2 Rho/ROCK pathway

The Rho protein family, as a class of small GTP-binding proteins, plays a crucial role as molecular switches in eukaryotic cells, finely regulating numerous signaling pathways (Uemura and Fukushima, 2021). Similarly, Rho-related kinase (ROCK) is an indispensable molecular switch in cells that controls basic life activities such as cell adhesion, proliferation, cytokine activation, migration of inflammatory cells, contraction of smooth muscle cells, and regulation of the cell cycle (Johan and Samuel, 2019). Previous studies have revealed an important interaction between Rho protein and the NF-κB signaling pathway, which can amplify inflammatory responses by enhancing the activity of the Rho/ROCK/NF-κB pathway (Huang et al., 2016).

The activation of the Rho/ROCK signaling pathway plays a key role in the pathological process of CI-AKI (Wang et al., 2018; Abbhi and Piplani, 2020). This pathway plays a crucial role in regulating renal vascular tone, maintaining cell survival, and remodeling the cytoskeleton. The activation of the Rho/ROCK pathway triggers the contraction of vascular smooth muscle, thereby reducing renal blood flow and affecting its filtration function. The contraction of renal blood vessels and the reduction of blood supply may be one of the key mechanisms underlying the occurrence of CI-AKI. Research has shown that ROCK inhibitors exhibit protective effects in various kidney injury models (Wang et al., 2018), as they can relax vascular smooth muscle, increase renal blood flow, and alleviate damage to renal tubular cells. Inhibiting the Rho/ROCK pathway can exert strong protective effects, including anti-inflammatory, anti-apoptotic, and antioxidant effects, as well as promoting vasodilation and enhancing endothelial function. Therefore, the use of ROCK inhibitors may represent a novel therapeutic approach for the prevention or treatment of CI-AKI. In a mouse model of CI-AKI, the activity of the Rho/ROCK pathway was increased, which ultimately led to enhanced NF-κB transcriptional activity, oxidative stress, inflammation, and apoptosis, thereby impairing kidney function (Wang et al., 2018). In the study by Su J, it was found that pretreatment with atorvastatin could alleviate the development of CI-AKI, potentially through the inhibition of the Rho/ROCK pathway and the reduction of apoptosis in renal tubular cells (Su et al., 2014).

## 4 Oxidative stress pathways

Oxidative stress is a key pathogenic mechanism of CI-AKI (Kusirisin et al., 2023). CM can induce the production of ROS and reduce the activity of antioxidant enzymes, leading to oxidative stress and kidney function damage (Kusirisin et al., 2020). Additionally, ROS can also damage mitochondrial and nuclear DNA, lipid membranes, and cellular proteins, causing apoptosis and necrosis.

### 4.1 NOXs pathway

Vascular NADPH oxidases (NOXs) are important transmembrane enzyme complexes that regulate the production of ROS in vascular cells, playing a crucial role in cellular redox homeostasis (Di Cristofano et al., 2021). Upon activation, NOX facilitates the transfer of electrons from NADPH to oxygen, forming superoxide radicals. NOX can also trigger the production of mitochondrial ROS, amplifying the impact of ROS (Bedard and Krause, 2007). The eNOS enzyme converts L-arginine and oxygen into L-citrulline and NO, but its excessive production can exacerbate oxidative stress (Karbach et al., 2014). Additionally, superoxide dismutase (SOD) plays a key role in scavenging excessive ROS (Ali et al., 2020). SOD converts superoxide anions into oxygen and hydrogen peroxide, which is then transformed into water by catalase, thereby counteracting oxidative stress.

Numerous studies on CI-AKI have unveiled that CM induces NOX activation, upregulates the expression of NOX4 and NOX2, heightens ROS production in renal tubular cells, and triggers apoptosis and necrosis. Conversely, the inhibition of NOX serves to mitigate oxidative stress in endothelial cells (Manda et al., 2019). Anomalies in NOX activation contribute to eNOS dysregulation and uncoupling, resulting in diminished NO bioavailability, decreased production, superoxide generation, and compromised or inactivated SOD. This establishes a detrimental cycle of oxidative stress, exacerbating endothelial dysfunction, diminishing NO release, and amplifying ROS production, ultimately leading to apoptosis in endothelial cells (Scoditti et al., 2013; Chen et al., 2020b). Furthermore, evidence indicates that pre-treatment with SOD significantly prevents CM-induced renal dysfunction (Pisani et al., 2014).

### 4.2 AMPK/PKC pathway

Adenosine monophosphate-activated protein kinase (AMPK) serves as a key regulator of biological energy metabolism, expressed in multiple metabolism-related organs and activated by various physiological stimuli. AMPK inhibits NOX in endothelial cells, which is crucial for maintaining redox homeostasis (Rodríguez et al., 2020). In the complex signaling transduction process, Protein kinase C (PKC) is a key effector molecule within the G protein-coupled receptor system.

Bioinformatics analysis has revealed that in CI-AKI, dysregulated miRNAs are likely to control the winged nut, transforming growth factor-β, and AMPK signaling pathways (Wang et al., 2019). In recent years, multiple studies have focused on the role of the AMPK/PKC pathway in CI-AKI. The study found that during the pathological process of CI-AKI, contrast media CM induced PKC phosphorylation events activate PKC, which not only controls the activation of NOX but also leads to the uncoupling of eNOS, thereby triggering oxidative stress and ultimately resulting in endothelial cell damage. The research indicates that the AMPK/PKC pathway plays a significant role in regulating the production of ROS and apoptosis. Tumor necrosis factor-α and hyperglycemia promote the release of ROS and apoptosis through a PKC-dependent NOX activation pathway, which plays a key role in the development of CI-AKI. Yan F’s research further confirms that PKC-dependent eNOS uncoupling is a core mechanism in inducing oxidative damage to endothelial cells (Yan et al., 2017). In addition, the inhibitory effect of D-4F, particularly on the production of ROS and the formation of peroxynitrite (ONOO-), is closely related to the AMPK/PKC pathway, which is of great significance for alleviating CM-induced apoptosis and inflammatory responses (Guo et al., 2021). The study also revealed that transient receptor potential ankyrin 1 maintains mitochondrial dynamics through the AMPK/Dynamin-1-like protein (DRP1) pathway, thereby providing protection for the kidneys and preventing contrast agent-induced tubular damage. This protective mechanism involves the regulation of mitochondrial fusion/fission-related protein expression and the reduction of mitochondrial fission by inhibiting AMP-activated protein kinase, thereby maintaining mitochondrial stability and function. These findings provide new targets and strategies for the treatment of CI-AKI, emphasizing the potential value of the AMPK/PKC pathway in kidney protection (Wang X. et al., 2024).

### 4.3 Nrf2/HO-1 pathway

Nuclear factor erythroid 2-related factor 2 (Nrf2) is widely present in most tissues, serving as both an antioxidant and a key regulator of oxidative stress and metabolism (Zuo et al., 2022). As a transcription factor, Nrf2 binds to the keap1 protein under normal conditions and translocates to the nucleus during oxidative stress, activating the transcription of antioxidant and detoxification enzymes to protect cells from damage caused by kidney diseases (Crisman et al., 2023). Heme oxygenase (HO) is the rate-limiting enzyme for heme degradation, and HO-1 is induced under oxidative stress, protecting the kidneys by scavenging free radicals, a protective effect that has been confirmed by research (Lianos and Detsika, 2023). Activation of Nrf2 also promotes the transcription of downstream signals such as HO-1, enhancing cellular protection (Feng et al., 2023).

The Nrf2 pathway has become a focal point of interest as a key protective target in the study of CI-AKI. A substantial body of research literature indicates that activating the Nrf2 pathway can effectively prevent the occurrence of CI-AKI (Khaleel et al., 2017; Xu et al., 2022). In the protective mechanism against CI-AKI, the Nrf2/HO-1 pathway plays a crucial role. The role of Nrf2 in CI-AKI has received widespread attention. In the study by Kim et al. (2019), using *Nrf2* knockout mice and NRK-52E cell models, researchers delved into the importance of Nrf2 in CI-AKI. The study found that when *Nrf2* function is absent, treatment with CM leads to an excessive production of ROS, inflammation, and increased apoptosis, whereas the activation of *Nrf2* significantly mitigates the damage to renal tubular cells. These results suggest that upregulating the Nrf2/HO-1 pathway can elicit cellular adaptive protective responses to counteract tissue damage, increased oxidative stress, and apoptosis caused by CM (Xu et al., 2022). Additionally, research has shown that epigallocatechin gallate significantly improves oxidative stress and inflammation in renal tissue by enhancing the Nrf2/HO-1 antioxidant pathway and inhibiting the NLRP3/IL-1β inflammatory pathway (Zhao et al., 2016). Among these protective mechanisms, HO-1 stands out as a key molecular target against CI-AKI. Notably, 2,2-dimethylthiazoline dihydrochloride upregulates Nrf2 expression by promoting the ubiquitination and degradation of Keap1. More significantly, the activated Nrf2/Slc7a11 pathway effectively inhibits the occurrence of ferroptosis.

### 4.4 Nrf2/SIRT3/SOD2 pathway

Sirtuin 3 (SIRT3), a nicotinamide adenine dinucleotide (NAD^+^)-dependent deacetylase located in the mitochondrial matrix, is involved in ATP production, oxidative stress response, and energy metabolism. Studies have found that SIRT3 plays a crucial role in maintaining mitochondrial function and preventing kidney damage under oxidative stress. In the kidneys, the activation of SIRT3 can convert acetylated SOD2 into its active form SOD2 to eliminate ROS. SIRT3 also regulates the transport of mitochondria through the microtubule network between renal tubular epithelial cells, maintaining cellular energy status and defending against oxidative stress. The transcription factor Nrf2 regulates SIRT3 expression by binding to the SIRT3 promoter through its NRF-2A subunit, affecting its levels (Castillo et al., 2019; Satterstrom et al., 2015).

The protective role of SIRT3 in a variety of kidney diseases has been extensively validated. Morigi M’s study (Morigi et al., 2015) demonstrated that the activation of SIRT3 can alleviate mitochondrial dysfunction in cisplatin-induced acute kidney injury. In the angiotensin II-induced kidney injury model, SIRT3 has been proven effective in preventing renal tubulointerstitial fibrosis, which is achieved by reducing oxidative stress and mitochondrial dysfunction (He et al., 2018). In the animal model of CI-AKI, exposure to CM significantly increased the expression of SIRT3 in wild-type mice and HK-2 cells, while the absence of SIRT3 exacerbated CI-AKI. Additionally, as a transcription factor, Nrf2 plays a key role in regulating the activation of the SIRT3/SOD2 signaling pathway; when Nrf2-silenced HK-2 cells were exposed to CM, they suffered severe cellular oxidative stress, with reduced expression of SIRT3 and SOD2, and an increased level of acetylated SOD2 (Zhang Q. et al., 2018; Zhou et al., 2019). Similarly, in the study by Zhang C and colleagues, the knockout of *Sirt3* and the use of specific *Sirt3* siRNA in mice with iothalamate-induced acute kidney injury and NRK-52E cells eliminated the renal protective effects of the medication, further confirming that activating *Sirt3* in CI-AKI models, both *in vitro* and *in vivo*, can provide protection against oxidative stress, apoptosis, and inflammation, potentially improving CI-AKI in clinical practice (Zhang et al., 2021).

### 4.5 SIRT1/PGC-1α/FoxO1 pathway

Sirtuin-1 (SIRT1) is an NAD^+^-dependent deacetylase involved in a variety of cellular functions, such as proliferation, DNA repair, and antioxidation. SIRT1 is widely expressed in tissues such as the kidneys and serves as a sensitive energy sensor (Chen et al., 2020c). Studies have shown that SIRT1 can prevent ischemia, resist inflammation, reduce ROS, and counteract cell death (Tang, 2016; Yang et al., 2022). Peroxisome proliferator-activated receptor-γ coactivator 1α (PGC-1α) is expressed in tissues with strong oxidative capacity and alleviates oxidative stress by enhancing SOD2 expression and reducing ROS (Tong et al., 2023).

**FIGURE 2:**
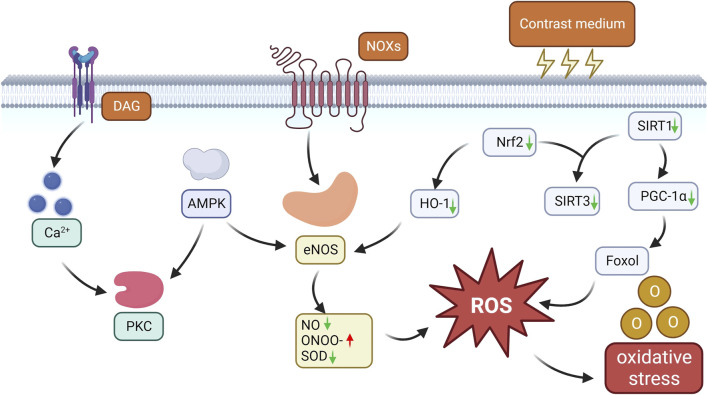
Oxidative stress pathways in CI-AKI. CM induces NOX activation and upregulates NOX4 expression, while aberrant NOX activation promotes eNOS dysregulation and uncoupling, leading to decrease NO bioavailability, superoxide production and ONOO-formation, and consequently aggravates endothelial dysfunction with decreased NO release and elevated ROS production. CM also induces PKC phosphorylation through the AMPK/PKC pathway, which controls NOX activation and eNOS uncoupling, leading to oxidative stress and endothelial damage. CM also reduces nrf2 expression, which inhibits HO-1 transcriptional activation, leading to an increase in eNOS production and prolonged oxidative stress. In addition, CM further increases ROS production by decreasing the expression of oxidative stress protective factors such as SIRT1, SIRT3, PGC-1α and dephosphorylated FoxO1, triggering oxidative stress.

SIRT1 regulates histones, transcription factors, and cofactors through deacetylation, thereby activating and controlling key pathways of the cellular stress response, including peroxisome proliferator-activated receptor γ coactivator-1α (PGC-1α). Therefore, the activation of SIRT1 and PGC-1α may provide kidney protection by reducing intracellular oxidative stress and apoptosis levels. In studies on CI-AKI, Hong Y′ s study has revealed the important protective role of the SIRT1-PGC-1α-Forkhead box O1 (FoxO1) signaling pathway (Hong et al., 2017). The study found that CM can downregulate the expression of SIRT1 and PGC-1α in both *in vivo* and *in vitro* experiments. However, activating SIRT1 with resveratrol can significantly reduce oxidative stress, inflammatory response, and apoptosis of renal tubular cells, and increase the expression levels of SIRT1, PGC-1α, and dephosphorylated FoxO1 in mouse kidneys. In addition, the study also found that astaxanthin has a protective effect against CI-AKI (Liu et al., 2018). *In vivo* model studies, the protective mechanism of astaxanthin is related to the SIRT1-p53 signaling pathway, which can effectively reduce the content of NO and 3-nitrotyrosine in CI-AKI renal tissue, thereby alleviating kidney damage caused by CM. *In vitro* experiments, astaxanthin inhibits oxidative stress and apoptosis through the SIRT1/FOXO3a pathway, thus reducing CI-AKI. Similarly, studies have also shown that quercetin can activate SIRT1, enhance the Nrf 2/HO-1/SOD1 signaling pathway, inhibit apoptosis and M1 macrophage polarization, and exert anti-inflammatory and antioxidant effects, thereby reducing contrast-induced acute kidney injury in type 1 diabetic mice (Wu et al., 2024) (Figure 2).

### 4.6 Interactions between oxidative stress and other pathways

Oxidative stress has a significant impact on cellular signaling pathways, especially in processes such as inflammation, apoptosis, and ferroptosis. These pathways interact with each other and jointly control cell survival and death. If the signals of oxidative stress are lost, other pathways will activate compensatory mechanisms to maintain the stability of the intracellular environment.

The interweaving of oxidative stress and inflammatory pathways forms a complex interactive network that exacerbates each other and drives the progression of various diseases. Oxidative stress can stimulate the activity of the transcription factor NF-κB, prompting inflammatory cells to release prostaglandins, cytokines, and chemokines, which further exacerbate the inflammatory response. In the case of weakened oxidative stress signals, this interaction can activate compensatory mechanisms to reactivate oxidative stress or compensate for its effects. Notably, myeloperoxidase can catalyze the generation of oxidants, such as hypochlorous acid, during inflammation, which can trigger oxidative stress even in the absence of direct oxidative stress signals (Daenen et al., 2019). In addition, cells can compensate for oxidative stress by enhancing the expression of inflammatory receptors such as Toll-like receptors and NOD-like receptors. However, in the field of CI-AKI research, although studies have shown that CM can simultaneously increase oxidative stress and inflammation levels, leading to kidney injury and renal function decline (Fu et al., 2023; Zhang et al., 2024), current research on the deeper interaction mechanisms between these two pathways is still limited.

There is also a complex interaction between oxidative stress and ferroptosis. Ferroptosis can be triggered by promoting lipid peroxidation under oxidative stress or inflammatory conditions. Oxidative stress may affect key enzymes regulating iron deposition, such as depletion and inactivation of antioxidant enzymes glutathione (GSH) and glutathione peroxidase 4 (GPX4), leading to disruption of the intracellular antioxidant defense system and reduced resistance to lipid peroxidation, thereby promoting ferroptosis. Oxidative stress affects iron metabolism, leading to intracellular iron accumulation. Interestingly, disturbances in iron metabolism can result in iron-catalyzed oxidation reactions, forming ROS or similar harmful substances to compensate for oxidative stress. Previous studies have shown that CM can directly inhibit the activity of antioxidant enzymes, such as SOD and glutathione peroxidase, indicating that exposure to CM may activate ferroptosis pathways even without direct oxidative stress activation signals, thereby triggering more severe oxidative stress. In the field of CI-AKI research, multiple findings have revealed that oxidative stress and ferroptosis often occur concurrently, with the Nrf2 pathway playing a crucial role in regulating ferroptosis. For instance, Gao Z’s research indicates that activating the HO-1/Nrf2 pathway upregulates the expression of GPX4, which can reduce CI-AKI and inhibit both oxidative stress and ferroptosis (Gao et al., 2022). However, this study did not further explore and supplement the interaction between these two mechanisms. Therefore, it is urgent to study the interaction mechanism between oxidative stress and ferroptosis in CI-AKI.

Oxidative stress can lead to mitochondrial dysfunction, regulate the activity of key proteins involved in apoptosis, release cytochrome c into the cytoplasm to activate the caspase cascade reaction, activate the DNA damage response pathway, and ultimately cause apoptosis. During apoptosis, mitochondrial function becomes impaired, which may lead to an increase in ROS production, further exacerbating oxidative stress (Yoshikawa and You, 2024). Additionally, during apoptosis, the expression and activity of intracellular antioxidant enzymes may be inhibited, thereby reducing the cell’s antioxidant capacity. These two pathways interact and cross-regulate each other. One study utilized kidney-targeted polymer nanoparticles to efficiently deliver siRNA to reduce the expression of the mitochondrial enzyme arginase-2(Arg-2) in renal tubules. In both *in vitro* and *in vivo* CI-AKI models, this approach not only diminished apoptosis resulting from mitochondrial dysfunction but also mitigated oxidative stress (Gu et al., 2024). Additionally, research has indicated that the mitochondrial fission inhibitor 1 can also reduce oxidative stress and ameliorate CI-AKI (Wang X. et al., 2024). These findings suggest that inhibiting mitochondrial dysfunction and fission may reduce oxidative stress and serve a protective role in CI-AKI, potentially emerging as a novel therapeutic strategy for preventing CI-AKI.

Oxidative stress can trigger endoplasmic reticulum stress (ERS) through various mechanisms, such as damaging protein folding enzymes and molecular chaperones in the endoplasmic reticulum, interfering with the function of endoplasmic reticulum calcium pumps, leading to an imbalance in calcium ion homeostasis, and ultimately triggering cell apoptosis. In cases of weakened oxidative stress signals, the unfolded protein response (UPR) activated by endoplasmic reticulum stress may promote the generation of ROS through various pathways, including activating NADPH oxidase or inhibiting the function of antioxidant enzymes. Meanwhile, there is cross-regulation between endoplasmic reticulum stress and oxidative stress; for example, activation of the c-Jun N-terminal kinase (JNK) pathway can promote ROS production. In the study of CI-AKI, researchers have found that CM can increase endoplasmic reticulum stress, reactive oxygen species production, and the expression of apoptotic proteins in rat kidney tissue (Liu Q. et al., 2023). However, in-depth research into the interaction between endoplasmic reticulum stress and other signaling pathways in CI-AKI is still lacking, and this area warrants further investigation.

Understanding the interaction between oxidative stress and other signaling pathways in CI-AKI is crucial for developing therapeutic strategies, but there is a lack of research in this area. It is hoped that further studies can help us target these interactions to reduce cell damage and death, and to prevent or alleviate CI-AKI.

## 5 Apoptosis pathways

Apoptosis is an evolutionary mechanism for removing excess cells to preserve individual physiology (Elmore, 2007). Cell survival is balanced by anti- and pro-apoptotic processes, with apoptosis signals mainly triggered by mitochondrial and endoplasmic reticulum stress (Li and Ren, 2020). This process is crucial in CI-AKI pathophysiology, as evidenced by DNA fragmentation and programmed cell death in various cell types in a CI-AKI mouse model.

### 5.1 Mitochondrial autophagy-mediated apoptosis pathways

Mitochondrial autophagy (Mitophagy) removes damaged mitochondria to preserve their health and acts as a defense against oxidative stress and inflammation, preventing mitochondrial-induced cell death (Yang et al., 2019). In CI-AKI, contrast agent-induced oxidative stress and mitochondrial damage trigger mitophagy to protect tissues (VanderVeen et al., 2017; Lin et al., 2019; Yang et al., 2018). CM exposure may cause protein misfolding and aggregation in renal tubules, leading to cell damage and death, potentially activating mitophagy. Excessive ROS from CM can also damage mitochondria, leading to membrane potential loss or fragmentation, which initiates mitophagy (Bae et al., 2020).

Parkin (PRKN), a member of the really interesting new gene family of E3 ubiquitin ligases, is crucial for mitochondrial quality control. PINK1, a mitochondrial kinase, typically undergoes cleavage and degradation. Damage that causes depolarization prevents the import and degradation of PINK1, leading to its accumulation on the outer membrane of damaged mitochondria and triggering mitophagy to eliminate these damaged mitochondria, resulting in a decrease in mitochondrial membrane potential (ΔΨm). The PINK1-PRKN-dependent mitophagy pathway exhibits a protective role in CI-AKI. Investigations have shown that the induced mitophagy in renal tubular epithelial cells depends on the PINK1-PRKN pathway, and silencing this pathway eliminates autophagy and exacerbates mitochondrial damage and apoptosis. Moreover, PINK1-PRKN-mediated mitophagy plays a protective role in CI-AKI by reducing mitochondrial ROS and the activation of NLRP3 inflammasomes (VanderVeen et al., 2017). Yang suggested that rapamycin exerts nephroprotective effects against CI-AKI by inducing robust mitophagy, thereby mitigating mitochondrial damage and oxidative stress (Yang et al., 2018). Bae reported that paricalcitol can confer renoprotective effects against CI-AKI through a PINK1-PRKN-dependent mitophagy pathway (Bae et al., 2020). Hypercholesterolemia exacerbates contrast agent-induced acute kidney injury, with the accumulation of oxidized low-density lipoprotein (Ox-LDL) acting as a cytotoxic factor. Studies indicate that Ox-LDL intensifies the damage to cells by increasing mitochondrial damage and oxidative stress responses. However, enhancing the level of PINK1/PRKN-dependent mitophagy can effectively mitigate this damage, suggesting that a deficiency in mitochondrial autophagy function may be associated with the aggravation of injury (Yang et al., 2023).

In addition to the PINK1-PRKN-dependent mitochondrial autophagy pathway, the protective roles of numerous mitochondrial autophagy pathways in CI-AKI are being gradually uncovered. CM treatment can upregulate the expression of miR-30e-5p in renal cells, subsequently triggering apoptosis by inhibiting the Beclin1-dependent autophagy pathway, which may be an important mechanism in the pathogenesis of CI-AKI (Liu et al., 2021). Studies have shown that stabilizing hypoxia inducible factor 1 alpha and activating its downstream BNIP3-mediated mitochondrial autophagy can effectively protect renal tubular epithelial cells from CM-induced damage both *in vitro* and *in vivo*. Moreover, the absence of Bcl-2/adenovirus E1B 19kDa interacting protein 3 (BNIP3) significantly reduces the level of mitochondrial autophagy and markedly exacerbates apoptosis and kidney damage, indicating that BNIP3-mediated mitochondrial autophagy plays a protective role in CI-AKI (Lin et al., 2021). Furthermore, the high expression of αKlotho protein in the kidneys has shown a positive effect in the treatment of CI-AKI, as it can protect the kidneys and HK-2 cells from contrast agent-induced damage by inhibiting the activation of the NLRP3 inflammasome and promoting autophagy (Zhu et al., 2021).

### 5.2 Bax/Bcl-2/caspase3, 9 pathway-mediated apoptosis pathway

The B-cell lymphoma 2 (Bcl-2) family of proteins finely regulates mitochondrial integrity, affecting outer membrane permeability and the apoptosis process (Czabotar and Garcia-Saez, 2023). This family is divided into anti-apoptotic and pro-apoptotic categories. Bcl-2 associated X protein (Bax) and Bcl-2 play a key role in mitochondrial damage during apoptosis, with Bcl-2 protecting the cell, while pro-apoptotic proteins promote cell death. Bax/Bcl-2 homologous antagonist/killer (Bak) are the key proteins causing mitochondrial damage. Caspase proteins are special proteases closely related to apoptosis, and the initiator caspase proteins activate other effector caspase proteins after receiving apoptotic signals, executing the apoptosis program (Xi et al., 2022).

Regarding CI-AKI, numerous studies have demonstrated that CM inhibits Bcl-2 expression, promotes the expression of pro-apoptotic member proteins, and increases the expression of caspase 3 and caspase 9, suggesting the involvement of the intrinsic pathway of CM-induced apoptosis of renal cells in CI-AKI (Yue et al., 2024; Quintavalle et al., 2011). Xu et al. (2022) observed that in the kidney tissues of CI-AKI rats after Tolvaptan pretreatment, the expression of pro-apoptotic proteins involved in cleaving caspase 3 and Bax, as well as mitochondrial fusion proteins DRP1 and Mitofusin 2, was downregulated, while the expression of Bcl-2 and PINK1 was upregulated (Xu et al., 2022). In a study by Huang S et al., the protein expression levels of Bax, caspase-3, Bcl-2, and cytochrome C (Cyt C) were examined. Kidney tissues from CI-AKI rats showed increased expression of Bax, caspase-3, and Cyt C and decreased expression of Bcl-2. Immunofluorescence analysis similarly revealed increased expression of Bax and caspase-3 in association with apoptotic cell death (Huang et al., 2020).

### 5.3 MAPK pathway-mediated apoptosis pathway

Mitogen-activated protein kinase (MAPK) is a class of protein kinases that are activated in response to a variety of external signals and are present in all eukaryotic cells (Quintavalle et al., 2011). Its signaling pathway consists of three sequentially activated kinases: mitogen-activated protein kinase kinase kinase (MAPKKK), mitogen-activated protein kinase kinase (MAPKK), and MAPK. This pathway is crucial for key processes such as cell growth, differentiation, stress response, and inflammatory reactions, and plays a significant role in the regulation of gene expression and cellular functions.

The activation of the MAPK pathway plays a crucial role in the pathogenesis of CI-AKI. Excessive ROS induced by CM can activate the MAPK signaling pathway through a series of cascade reactions involving four subfamilies, including the extracellular signal-regulated kinase (ERK), JNK, and p38 pathways. These pathways contribute to the activation of caspase-9 and caspase-3, thereby triggering apoptosis. Furthermore, it has been observed that the ROS/apoptosis signal-regulating kinase 1 (ASK1) signaling pathway is involved in the apoptotic process of CI-AKI (Yuan et al., 2022). ROS-induced apoptosis is dependent on ASK1, which belongs to the MAPKK family. Various stresses can activate ASK1, and the ROS/ASK1 signaling pathway can activate JNK and p38 MAPK to enhance the intrinsic apoptotic pathway. This pathway serves as a key signaling route in cytokine- and stress-induced apoptosis. Studies by Quintavalle have demonstrated that CM induces apoptosis in renal cells by increasing intracellular ROS levels through the activation of the JNK 1/2 and p38 pathways (He et al., 2016). CM was shown to increase pro-apoptotic members, and pretreatment with JNK 1/2 and p38 inhibitors reduced CM-induced upregulation of pro-apoptotic members of the Bcl-2 family and caspase-3 activation. These findings underscore the critical role of the MAPK pathway in intrinsic apoptotic signaling in CI-AKI. Furthermore, Andreucci revealed that in HK-2 cells, CM induced phosphorylation of p38 MAPK, JNK, and NF-κB, ultimately leading to inflammation and apoptosis (Andreucci et al., 2011).

### 5.4 PI3k/Akt/mTOR/Nrf2 pathway-mediated apoptotic pathway

Phosphatidylinositol 3-kinase (PI3K), an enzyme inside cells with dual kinase activities, is triggered by signals from tyrosine kinases and G protein-coupled receptors. It moves near the cell membrane, where it helps Akt move to the membrane and get activated. Active Akt then phosphorylates Mechanistic Target of Rapamycin (mTOR), negatively regulating the PI3K/Protein kinase B (Akt) pathway, which slows cell growth and induces apoptosis (Margaria et al., 2020; Zhang and McCullough, 2016).

An increasing body of research evidence indicates that the PI3K/Akt signaling pathway plays a significant role in the apoptosis process of CI-AKI (Xie et al., 2017; Tongqiang et al., 2016; Hu et al., 2017; Liu et al., 2015). Study results show that enhancing the PI3K/Akt signaling pathway can significantly mitigate the apoptotic effects of CM on HK-2 cells, as well as the renal vasoconstriction induced by CM. Additionally, salvianolic acid B has been proven to be an effective preventive measure, protecting against CM-induced cellular and tissue damage by activating the PI3K/Akt signaling pathway. This activation process is accompanied by the upregulation of antioxidant and detoxification enzymes, as well as the induced expression of Nrf2 and HO-1 (Tongqiang et al., 2016). Moreover, the protective effect of the PI3K/Akt pathway is closely related to the phosphorylation activation of GSK-3β, which promotes the expression of nuclear Nrf2 and reduces the levels of nuclear NF-κB. The use of specific inhibitors of Glycogen synthase kinase-3β (GSK-3β) can inhibit the opening of the mitochondrial permeability transition pore by activating Nrf2 and inhibiting NF-κB, thereby reducing oxidative stress and inflammatory responses.

### 5.5 Endoplasmic reticulum stress-mediated apoptotic pathways

The endoplasmic reticulum is sensitive to environmental changes and can accumulate unfolded proteins, disrupting Ca^2+^ balance and causing ERS (Liu et al., 2022). During ERS, the body responds with a stress mechanism mediated by GRP78 and sensors like endoplasmic reticulum kinase (PERK), inositol-requiring enzyme 1α (IRE1α), and activating transcription factor 6 (ATF6). Normally, these sensors are inactive when bound to GRP78, but ERS causes them to separate, enabling GRP78 to clear misfolded proteins. Severe or prolonged ERS can activate apoptosis pathways through these sensors to remove damaged cells (Iurlaro and Muñoz-Pinedo, 2016). ERS-induced apoptosis is key in kidney diseases like CI-AKI (Cao et al., 2014; Hetz, 2012) and is triggered by CM exposure, which affects survival or apoptosis signals in renal tubular cells.

#### 5.5.1 IRE1/PERK/CHOP pathway

CHOP, a C/EBP family member, governs cell function and apoptosis genes. ERS induces CHOP via PERK, ATF6, and IRE1, promoting apoptosis. IRE1 activates TNF receptors and ASK1, JNK, p38 MAPK pathways. JNK phosphorylation aids apoptosis via Bcl-2 proteins; its inhibitor SP600125 blocks CHOP, highlighting JNK’s role. ERS also boosts eIF2α phosphorylation through PERK, raising ATF4 and CHOP, triggering apoptosis.

The IRE1 pathway, a central component of the unfolded protein response, activates tumor necrosis factor-associated receptors, as well as downstream ASK1 and the JNK and p38 MAPK pathways (Kim et al., 2018). Notably, JNK phosphorylation regulates the expression of Bcl-2 family members, promoting the activation of pro-apoptotic genes and suppressing anti-apoptotic proteins. Additionally, studies have demonstrated that the JNK inhibitor SP600125 prevents CHOP upregulation and death receptor 5 expression, suggesting the involvement of JNK activation in CHOP regulation (Guo et al., 2017). Moreover, during ER stress, PERK enhances the phosphorylation of Eukaryotic Initiation Factor 2 alpha (eIF2α), which boosts the translational activity of activating transcription factor 4 (ATF4). Consequently, ATF4 induces the upregulation of CHOP. The PERK-eIF2α-ATF4-CHOP pathway triggers apoptosis by interacting with death receptor pathway proteins (Cao et al., 2012; Taniguchi and Yoshida, 2015). CHOP, a member of the CCAAT/Enhancer Binding Protein (C/EBP) family, plays a crucial role in regulating genes associated with proliferation, differentiation, expression, and energy metabolism (Hu et al., 2019). When ERS occurs in cells, the three pathways of PERK, ATF6, and IRE1 can regulate the CHOP gene, significantly increasing the expression of CHOP proteins, activating several upstream pro-apoptotic signals, and acting on the mitochondrial membrane, which further activates downstream caspase family proteins (Wu et al., 2020). As a transcription factor, CHOP governs the expression of various anti-apoptotic and pro-apoptotic genes. It downregulates Bcl-2 while upregulating the expression of Bak and Bax, eventually leading to apoptosis (Tuzlak et al., 2016).

In our previous study, we observed that salvianolic acid B mitigated the expression of pro-apoptotic proteins CHOP and JNK, as well as the ERS initiator protein GRP78, and its downstream factors p-IRE1α and eIF-2α, to some extent in HK-2 cells exposed to CM. This suggests that salvianolic acid B may inhibit CM-induced apoptosis in HK-2 cells through the inhibition of the IRE1/ASK1/JNK pathway and the PERK/p-IRE1α/ATF4/CHOP pathway (Dong et al., 2021). Additionally, valsartan demonstrated potential nephroprotective effects against CM-induced renal cell apoptosis by inhibiting ERS. The study revealed that CM induced apoptosis through the activation of GRP78, ATF4, caspase-12, CHOP, and JNK (Peng et al., 2015).

#### 5.5.2 Ca^2+^/Casepase12 pathway

GRP78 is a key Ca^2+^-binding protein that regulates Ca^2+^ transport and unfolded protein responses, maintaining ER homeostasis. Its interaction with ERS sensors controls Ca^2+^ efflux. GRP78, binding to misfolded proteins, activates CHOP, which in turn regulates caspase-12, Bcl-2 proteins, and caspase-3, initiating apoptosis via ERS.

Regarding CI-AKI, intracellular calcium overload is recognized as a key contributor to ischemic cell injury and CI-AKI. Studies have linked both contrast-induced renal vasoconstriction and renal tubular cell apoptosis to alterations in calcium physiology (Yang et al., 2013a; Yang et al., 2013b). Additionally, the overproduction of ROS induced by CM is implicated in triggering ERS, leading to cytoplasmic Ca^2+^ overload. This intracellular Ca^2+^ overload may, in turn, contribute to increased ROS production, activation of p38 MAPK, and apoptosis in CI-AKI (Yang et al., 2013a). Calcium channel blockers have been identified as agents capable of reversing acute hemodynamic changes induced by contrast administration and mitigating CI-AKI. Yang, D’s animal study confirmed that tail vein injection of the Na^+^/Ca^2+^ exchanger reversed CM-induced endothelin-1 overproduction and renal vasoconstriction (Yang et al., 2013b). Furthermore, Ward observed an upregulation of caspase-12 in HK-2 cells exposed to CM (Ward et al., 2019). However, pretreatment of HK-2 cells with calcium concentration modulators protected against CM-induced mitochondrial toxicity, underscoring the pivotal role of calcium dysregulation in CI-AKI.

## 6 Ferroptosis pathway

Ferroptosis is a distinctive form of cell demise characterized by the accumulation of lipid ROS and iron due to the inactivation of GPX4. Cells undergoing ferroptosis exhibit distinctive features, including mitochondrial shrinkage, heightened membrane density, and rupture of the outer membrane, while the nuclear morphology remains unaltered (Jiang et al., 2021). Lipid peroxidation stands out as the primary hallmark of ferroptosis, with iron and ROS playing pivotal roles in prompting this ferroptosis. ROS generation serves as the central mechanism in CI-AKI. Exposure to CM elevates ROS levels, initiating apoptosis through lipid peroxidation, which, in turn, results in glutathione depletion and inactivation of the phospholipid peroxidase GPX4.

Recent investigations have underscored the crucial role of ferroptosis in kidney injury (Belavgeni et al., 2020; Martin-Sanchez et al., 2017; Deng Y. H. et al., 2023; Fang et al., 2021; Dai et al., 2023; Wang et al., 2024a). In a proteomics-based study, 604 different proteins were identified in renal tissues from the CI-AKI model, involving proteins associated with ferroptosis (Deng Y. H. et al., 2023). It is worth noting that in existing CI-AKI studies, ferroptosis often occurs in conjunction with the activation of oxidative stress pathways, and the degree of ferroptosis correspondingly decreases when the level of oxidative stress is reduced. Research has shown that maintaining the stability of SIRT1 expression can increase the expression level of GPX4, thereby reducing the occurrence of ferroptosis (Fang et al., 2021). In addition, Dai B’s study found that the ubiquitination and degradation of Keap1 increased, promoting the upregulation of Nrf2. The activated Nrf2/Scc7a11 pathway led to increased levels of GSH and GPX4, thereby inhibiting ferroptosis and alleviating CI-AKI (Dai et al., 2023). Wang W’s research further indicates that increasing the expression of FoxO3a and Nrf2 can effectively reduce cell apoptosis and ferroptosis induced by CM, confirming the protective role of the Akt/FoxO3a/Nrf2 signaling pathway in ferroptosis (Wang et al., 2024a). These research findings clearly indicate that the Nrf2-mediated signaling pathway plays an important protective role against ferroptosis in CI-AKI, and the interaction mechanism between oxidative stress and ferroptosis involved in this pathway may become a key target for the treatment of CI-AKI.

Notably, ferrostatin-1 (Fer-1) and deferoxamine (DFO), as inhibitors of ferroptosis and iron chelators, primarily regulate iron metabolism and redox balance to inhibit ferroptosis. Their renal protective effects have been preliminary validated (Xie et al., 2022; Wang et al., 2022; Deng et al., 2019). In a study on CI-AKI, Zhu Z found that damage to renal tubular cells was closely related to ferroptosis, both *in vivo* and *in vitro*, manifested by characteristic changes of ferroptosis, such as Fe^2+^ accumulation, lipid peroxidation, and decreased GPX4 activity (Zhu et al., 2024). Fer-1 and DFO not only enhance cell viability and reduce intracellular ROS production by inhibiting ferroptosis but also effectively alleviate damage to renal tubular cells in CI-AKI by suppressing iron overload and lipid peroxidation. These research findings reveal that targeted therapy against ferroptosis may become a promising therapeutic strategy for treating CI-AKI.

Ferroptosis is a special form of iron-related death that is characterized by being closely related to iron-catalyzed lipid peroxidation reactions and has specific metabolic pathways, inhibitory mechanisms, and morphological signs (Chen X. et al., 2020). However, iron metabolism disorders are not limited to ferroptosis but may also trigger other types of cell death. For example, when iron is overloaded, multiple cell death pathways such as apoptosis, necrosis, and ferroptosis can be triggered. In the oxidative stress pathway, iron generates free radicals by participating in redox processes such as the Fenton reaction, thereby exacerbating oxidative stress. Although the apoptotic process does not directly depend on iron, iron overload can induce apoptosis through the generation of free radicals and oxidative stress (Mithila et al., 2023). As a regulated form of necrosis, necroptosis does not depend on classical apoptotic pathways (such as caspases), while iron-catalyzed oxidative stress can promote cell membrane damage and leakage of cell contents. In the state of iron deficiency, the reduction of antioxidant enzyme activity (such as iron-sulfur cluster proteins) leads to increased oxidative stress and affects mitochondrial function, thereby activating apoptotic signaling pathways. Long-term iron deficiency may lead to energy metabolism disorders, cell swelling and membrane integrity damage, and ultimately cell necrosis. Iron deficiency may also activate the autophagy pathway as a survival strategy for cells to cope with nutritional deficiency, but excessive autophagy may also lead to cell death (Sung et al., 2023). In addition, abnormal iron metabolism may lead to lysosomal instability and trigger lysosome-dependent cell death, which may overlap with ferroptosis because lysosomal rupture releases iron ions and exacerbates lipid peroxidation.

These iron-related cell death pathways play a role in different physiological and pathological states, and the interaction and conversion between them are key points in the field of cell death research. Therefore, in addition to ferroptosis, abnormal iron metabolism may further aggravate the condition of CI-AKI by enhancing oxidative stress, promoting apoptosis, exacerbating inflammatory responses, and interfering with cell metabolism. At present, relevant research is still blank, therefore, a deep understanding of these pathways is crucial for the development of therapeutic strategies for CI-AKI.

## 7 Targeted drug delivery technologies and specific therapeutic targets

How to prevent and treat CI-AKI is a central focus of clinical research, but current methods such as hydration therapy and statin medication have limitations in efficacy, particularly in high-risk patients with cardiovascular disease. In response to these challenges, this review will refocus its attention on advanced targeted drug delivery technologies and research into specific therapeutic targets for CI-AKI, with the aim of finding strategies that can effectively protect the kidneys from CI-AKI-related damage.

### 7.1 Targeted drug delivery technologies

Targeted drug delivery technology is pivotal in the management of kidney diseases, including CI-AKI. This technology precisely targets damaged or diseased regions of the kidney, effectively minimizing systemic side effects, boosting treatment efficacy, and navigating through the kidney’s natural barriers with ease. Moreover, it substantially mitigates the risk of kidney damage from nephrotoxic agents. Specifically, the nanotechnology-based renal tubule-targeted drug delivery approach has paved a novel route for AKI treatment. Many drugs struggle to target renal tubular epithelial cells due to the constraints of the glomerular filtration barrier. However, research has demonstrated that 100-nm-diameter poly (lactic-co-glycolic acid) (PLGA) nanoparticles can selectively accumulate in the kidneys of mice with damage. The high accumulation of PLGA nanoparticles in ischemia-reperfusion kidneys, coupled with their renal tubule targeting, facilitates the delivery of therapeutic agents. Experimental data indicate that nanotechnology-mediated drug delivery for treating AKI and fibrosis is a promising approach (Yu et al., 2019). Additionally, targeted delivery systems utilizing other vectors, such as those carrying p53-related siRNA, are capable of effectively modulating apoptosis, metabolic, and inflammatory pathways, significantly alleviating kidney damage, apoptosis, and the infiltration of macrophages and neutrophils, thus enhancing renal function (Tang et al., 2022).

Building on previous research, Gu X’s team has developed a novel type of kidney-targeted polymer nanoparticle designed to deliver siRNA effectively, with the aim of reducing Arg-2 expression in renal tubules and assessing its potential in preventing CI-AKI (Gu et al., 2024). These nanoparticles employ a layer-by-layer assembly process, beginning with a coating of cationic chitosan on the PLGA core, followed by a layer of hyaluronic acid to prevent siRNA leakage. To improve kidney targeting, the team introduced kidney-targeting peptides to the outer layer of the hyaluronic acid. Twenty-4 h post-injection, these nanoparticles had accumulated within the kidneys and renal tubular cells, demonstrating their ability to reduce oxidative stress, restore mitochondrial function, and decrease apoptosis, thereby offering a promising therapeutic approach for the prevention of CI-AKI.

Targeted drug delivery technology has demonstrated significant potential in the management of CI-AKI. This innovative strategy is well-suited to tackle the challenges associated with CI-AKI. The precision of targeted drug delivery enables more customized and potent treatment approaches by specifically targeting the affected renal tissue. This ensures that the therapeutic agent is delivered directly to the injury site, reducing the exposure of healthy tissue to the medication while enhancing its therapeutic impact. Employing this targeted method not only diminishes the risk of adverse effects but also enhances the overall therapeutic efficacy index.

### 7.2 Specific therapeutic targets

The precise identification of specific therapeutic targets enables the refinement of treatment strategies, and by intervening at critical points in the pathogenesis of CI-AKI, the efficacy of treatments has been markedly enhanced. Moreover, targeted treatment approaches are anticipated to effectively slow the progression of kidney damage by selectively inhibiting pathways such as inflammatory responses and oxidative stress. As research delves deeper into the intricate molecular mechanisms of CI-AKI, the emergence of new targeted therapies suggests that patients will have access to more favorable treatment outcomes.

In the realm of CI-AKI research, the activation of Nrf2 is broadly recognized as a pivotal protective target. Nrf2 activation has the capacity to modulate a spectrum of antioxidant proteins, including SIRT1, SIRT3, and HO-1, and can also inhibit the process of ferroptosis. This enhances the antioxidant response, mitigates oxidative stress induced by contrast agents, and shields the kidneys from harm. A multitude of drugs, such as quercetin, lansoprazole, tolvaptan, sulforaphane, and 2,2-dimethylthiazolidine hydrochloride, are known to effectively stimulate the Nrf2 pathway (Feng et al., 2023; Khaleel et al., 2017; Xu et al., 2022; Kim et al., 2019; Zhao et al., 2016). Concurrently, the PI3K/Akt pathway is instrumental in the anti-apoptotic defenses against CI-AKI. Its activation serves to decrease apoptosis in renal tubular epithelial cells and to lower the generation of inflammatory mediators by activating Nrf2 and repressing NF-κB activity, thus offering protection against CI-AKI. Research indicates that both salvianolic acid B and O-linked β-N-acetylglucosamine can activate the Nrf2 and PI3K/Akt pathways, safeguarding the kidneys from CI-AKI (Tongqiang et al., 2016; Hu et al., 2017). Moreover, mitophagy, as a form of selective autophagy, manages mitochondrial integrity and ROS levels by eliminating damaged mitochondria. Studies have revealed that mitochondrial agonists can diminish the activation of NLRP3 inflammasomes through the PINK1-Parkin-mediated mitophagy route, thereby assuming a protective function in the context of CI-AKI.

CM induces kidney damage through the activation of inflammatory pathways. Consequently, the use of inflammatory inhibitors or drugs that target specific molecules can help to diminish the inflammatory response, thereby safeguarding the kidneys. Research has demonstrated that SGLT2 inhibitors can serve as a potential therapeutic strategy for preventing CI-AKI, owing to their inhibitory effects on the HIF-1α/HE4/NF-κB pathway (Huang et al., 2022). In a similar vein, inhibitors of the Rho pathway can selectively inhibit inflammation and shield the kidneys from CI-AKI. The application of GSK484 and DNase I to prevent the accumulation of neutrophil extracellular traps (NETs) *in vivo* or to facilitate their degradation can mitigate damage to renal vascular endothelial cells in CI-AKI mice, reverse pathological changes in the kidneys, and lower creatinine levels. The inhibition of NETs also reduces apoptosis and pyroptosis in renal cells and curtails the *in vivo* inflammatory response (Wang et al., 2023). Pharmacological research indicates that drugs such as acetylbritannilactone, baicalin, atorvastatin, and salvianolic acid B effectively protect against CI-AKI by targeting inflammatory pathways.

Furthermore, certain target proteins that engage with multiple signaling pathways are pivotal in the prevention and treatment of CI-AKI. Take high mobility group box 1 (HMGB1), for instance, which is a damage-associated molecular pattern molecule released by damaged cells or activated immune cells into the extracellular environment, where it binds to receptors such as Toll-like receptors and advanced glycation end product receptors. Research indicates that HMGB1 serves as a critical target for intervention in the treatment of CI-AKI; during the onset of CI-AKI, HMGB1 is expressed in renal tubular epithelial cells, macrophages, endothelial cells, and glomerular cells, contributing to the pathogenesis of various kidney diseases through the activation of its receptors (Mo et al., 2024).

Investigations into these targets open avenues for the development of novel treatment strategies aimed at diminishing the incidence and severity of CI-AKI. Nevertheless, the exploration of key targets in CI-AKI continues to encounter significant challenges. For instance, studies on ferroptosis targets predominantly depend on the activation of Nrf2, with a shortfall in detailed research on individual targets. Similarly, research into endoplasmic reticulum stress is lacking in the identification of specific target pathways and key target proteins.

## 8 Conclusion

**FIGURE 3:**
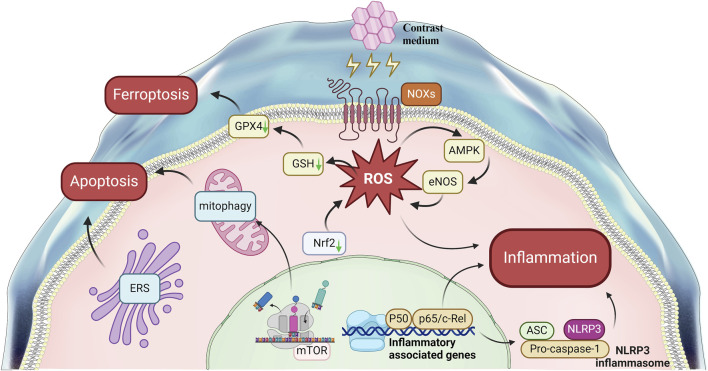
Signal Pathways Involved in contrast-induced acute kidney injury. This figure summarizes the main pathways Involved in the mechanism of CI-AKI.

CI-AKI is closely associated with a range of serious clinical consequences, including elevated mortality rates, extended hospitalizations, the requirement for renal replacement therapy, and an increased incidence of major adverse cardiac events. The precise pathogenesis of CI-AKI remains incompletely understood. The disease involves signaling pathways that are intimately linked to various biological processes, such as apoptosis, inflammation, oxidative stress, and ferroptosis (Figure 3). While research into CI-AKI-related pathways has been substantial, the exploration of ferroptosis and endoplasmic reticulum stress is less comprehensive and warrants further investigation. Additionally, the current understanding of the interplay between different signaling pathways in CI-AKI is limited, and there is an urgent need for more detailed molecular mechanism research to uncover the potential interactions between these pathways and to understand how they collectively contribute to the onset of CI-AKI.

Through these research avenues, we can achieve a more thorough understanding of the pathophysiology of CI-AKI and pinpoint specific targets within signaling pathways and their interactions, offering key insights for developing effective and targeted combination therapies. For instance, the application of kidney nano-targeted delivery technology to selectively activate Nrf2 has the potential to target multiple pathways, thereby offering protection against CI-AKI. Such research endeavors are poised to furnish more robust prevention and treatment strategies for future clinical use, ultimately enhancing patients’ clinical outcomes.
